# A Qualitative Evaluation of the Acceptability of an Interactive Voice Response System to Enhance Adherence to Isoniazid Preventive Therapy Among People Living with HIV in Ethiopia

**DOI:** 10.1007/s10461-016-1432-8

**Published:** 2016-05-24

**Authors:** Amrita Daftary, Yael Hirsch-Moverman, Getnet M. Kassie, Zenebe Melaku, Tsigereda Gadisa, Suzue Saito, Andrea A. Howard

**Affiliations:** 1 ICAP, Mailman School of Public Health, Columbia University, 722 West 168th Street, 13th Floor, New York, New York 10032 USA; 2 Centre for the AIDS Programme of Research in South Africa (CAPRISA), Nelson R. Mandela School of Medicine, University of KwaZulu Natal, Durban, South Africa; 3 Dalla Lana School of Public Health, University of Toronto, Toronto, Canada; 4 Department of Epidemiology, Mailman School of Public Health, Columbia University, New York, USA; 5 School of Public Health, Addis Ababa University, Addis Ababa, Ethiopia

**Keywords:** Interactive voice response, HIV/AIDS, Mobile health, Patient acceptability, Tuberculosis prevention, Qualitative methods

## Abstract

Interactive voice response (IVR) is increasingly used to monitor and promote medication adherence. In 2014, we evaluated patient acceptability toward IVR as part of the ENRICH Study, aimed to enhance adherence to isoniazid preventive therapy for tuberculosis prevention among HIV-positive adults in Ethiopia. Qualitative interviews were completed with 30 participants exposed to 2867 IVR calls, of which 24 % were completely answered. Individualized IVR options, treatment education, and time and cost savings facilitated IVR utilization, whereas poor IVR instruction, network and power malfunctions, one-way communication with providers, and delayed clinic follow-up inhibited utilization. IVR acceptability was complicated by HIV confidentiality, mobile phone access and literacy, and patient-provider trust. Incomplete calls likely reminded patients to take medication but were less likely to capture adherence or side effect data. Simple, automated systems that deliver health messages and triage clinic visits appear to be acceptable in this resource-limited setting.

## Resumen

La respuesta de voz interactiva (IVR por sus siglas en inglés) se utiliza cada vez más para monitorear y promover el cumplimiento del medicamento. En 2014, evaluamos la aceptación del paciente a la respuesta de voz interactiva (IVR) como parte del Estudio ENRICH, con el objetivo de mejorar el cumplimiento de la terapia preventiva de isoniacida para la prevención de la tuberculosis entre los adultos positivos al VIH en Etiopía. Las entrevistas cualitativas fueron completadas con 30 participantes expuestos a llamadas 2867 IVR, de las cuales 24 % fueron respondidas completamente. El acceso al teléfono, las opciones de la respuesta de voz interactiva (IVR), la educación al tratamiento y el ahorro en tiempo y costo, facilitaron la utilización de la respuesta de voz interactiva (IVR) del paciente, aunque la poca instrucción de la IVR, el mal funcionamiento de la energía y de la red, la comunicación unidireccional con los proveedores y el seguimiento clínico demorado, limitaron la utilización. La aceptación de la respuesta de voz interactiva (IVR) fue complicada por la confidencialidad del VIH, el acceso al teléfono móvil y la alfabetización, así como la confianza proveedor-paciente. Las llamadas incompletas probablemente recordaron a los pacientes tomar el medicamento, sin embargo, fue menos probable recoger los datos de cumplimiento o de efectos secundarios. Los sistemas simples y automatizados que transmiten los mensajes de salud y las visitas de triaje a la clínica parecen ser aceptados en este entorno de recursos limitados.

## Introduction

Despite the global scale-up of antiretroviral therapy (ART), tuberculosis (TB) remains the leading opportunistic infection and cause of mortality among people living with HIV (PLHIV), accounting for 26 % of HIV-related deaths worldwide [1, 2]. In 2014, an estimated 1.2 million new TB cases notified globally were HIV-coinfected; 74 % of these were in Africa [3]. Ethiopia ranks tenth among high TB-burden countries, with an estimated TB incidence of 207 per 100,000 [3]. Approximately 760,000 people were living with HIV in Ethiopia in 2012 [4].

HIV infection greatly increases the risk of developing TB, which may result from reactivation of latent TB infection or rapid progression to disease after recent infection [5]. Isoniazid preventive therapy (IPT) has been shown to reduce TB incidence in PLHIV not on ART [6]; IPT also reduces TB incidence [6] and the risk of death in patients on ART [7]. The World Health Organization (WHO) strongly recommends that PLHIV receive at least 6 months of IPT as part of a comprehensive package of HIV care, regardless of ART status [8]. Limited data from high-burden, resource-limited countries, however, suggest that adherence to IPT is suboptimal. In the WHO ProTEST project, involving IPT provision after HIV counseling and testing in Malawi, South Africa, and Zambia, only 24–59 % of patients completed the recommended course of IPT [9]. In a more recent review of studies from Uganda, South Africa, and Botswana, 47–88 % of patients completed IPT [10]; however, patients more likely to experience adherence challenges, such as those who were non-adherent to therapies for other chronic conditions, had experienced an adverse effect to IPT, and/or lived further away from clinics, were excluded from studies reporting higher rates [11–13]. There is an acute need to design and evaluate interventions that effectively address IPT adherence challenges so that the survival gains made with global ART scale-up are not undermined by the devastating impact of TB in PLHIV.

Mobile health (m-health), or the remote delivery of health care via mobile phone communication is a promising means of supporting and monitoring adherence to medication [14]. Mobile technology has the advantage of covering rural populations with poor transportation infrastructure in resource-limited settings [15]. The number of mobile phone subscriptions in sub-Saharan Africa is rising more rapidly than anywhere else in the world; the increase from 89 million in 2005 to an estimated 635 million by the end of 2014 [16] allows for innovative use of m-health technologies such as short-text messaging service (SMS) and interactive voice response (IVR) to automate health messaging and data collection.

The application of IVR to scientific research is becoming increasingly popular [17]. IVR data collection combines computerized self-interviewing with touch-tone telephone technology, allowing investigators to track participants and gather data without direct interviewer contact or deployment of complex, expensive equipment [17, 18]. Participants respond to automated questions by pushing numbers on their telephone keypad; their responses, recorded on a server connected to a phone network, may be used in real time to target interventions to those who are nonadherent [18]. IVR was recently tested as an intervention tool to increase adherence to ART in Uganda, and showed a high level of patient interest and participation [18, 19]. An important advantage over SMS is that IVR systems do not require participants to be literate. They are particularly suited to patients in Ethiopia, where the adult literacy rate is estimated to be 39 % [20].

As part of the **EN**hance Initiation and **R**etention in **I**PT **C**are for **H**IV (ENRICH) Study, we employed IVR as a tool to enhance adherence to IPT among adults living with HIV in Ethiopia. This paper describes a qualitative evaluation of patient acceptability toward IVR to inform its implementation in our study setting.

## Methods

For this evaluation, we used qualitative methods [21] to explore patient experiences with IVR technology and assess their acceptability toward IVR. The evaluation was nested within the parent ENRICH Study (https://clinicaltrials.gov NCT01926379), an implementation science cluster-randomized trial aimed to evaluate the effectiveness of a combination intervention package (CIP) versus standard of care, to improve initiation, adherence, and completion of a six-month course of IPT among HIV-positive patients newly enrolled in HIV care at ten urban primary health centers in Dire Dawa and Harari, Ethiopia. The CIP was implemented at five of these health centers since July 2013. Among several programmatic, structural, and psychosocial components, the ENRICH CIP incorporated real-time adherence support, delivered to patients by IVR technology in conjunction with a study-issued mobile phone, SIM card, and airtime vouchers. The costs associated with provision of a mobile phone, SIM card and 6 months’ airtime to facilitate IVR calls were approximately USD 45 per patient.

### IVR System Specifications

Grameen Foundation (http://www.grameenfoundation.org) developed the IVR system using an open-source MOTECH Suite application and managed the gateway for the IVR system over the local phone network. Calls customized to meet project needs and recorded by local radio personalities in four indigenous languages were placed through the Ethio Telecom mobile phone network (www.ethiotelecom.et). Health care workers utilized a tablet application developed by Commcare (http://www.commcarehq.org) to register patients’ phones to receive calls according to specified timing; this information was transmitted to MOTECH via the 2G cellular network.

### IVR Call Algorithm

**Fig. 1:**
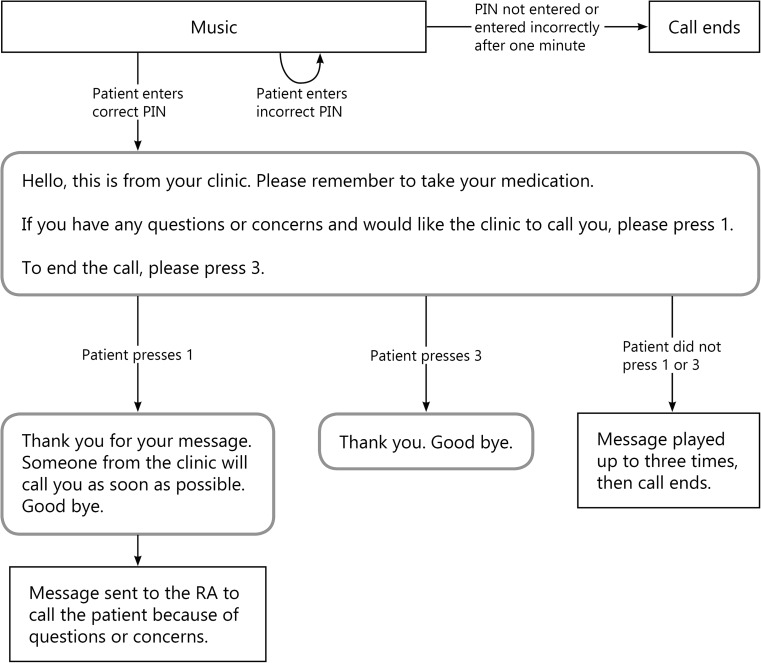
Example of IVR flow chart for medication adherence reminder call

**Fig. 2:**
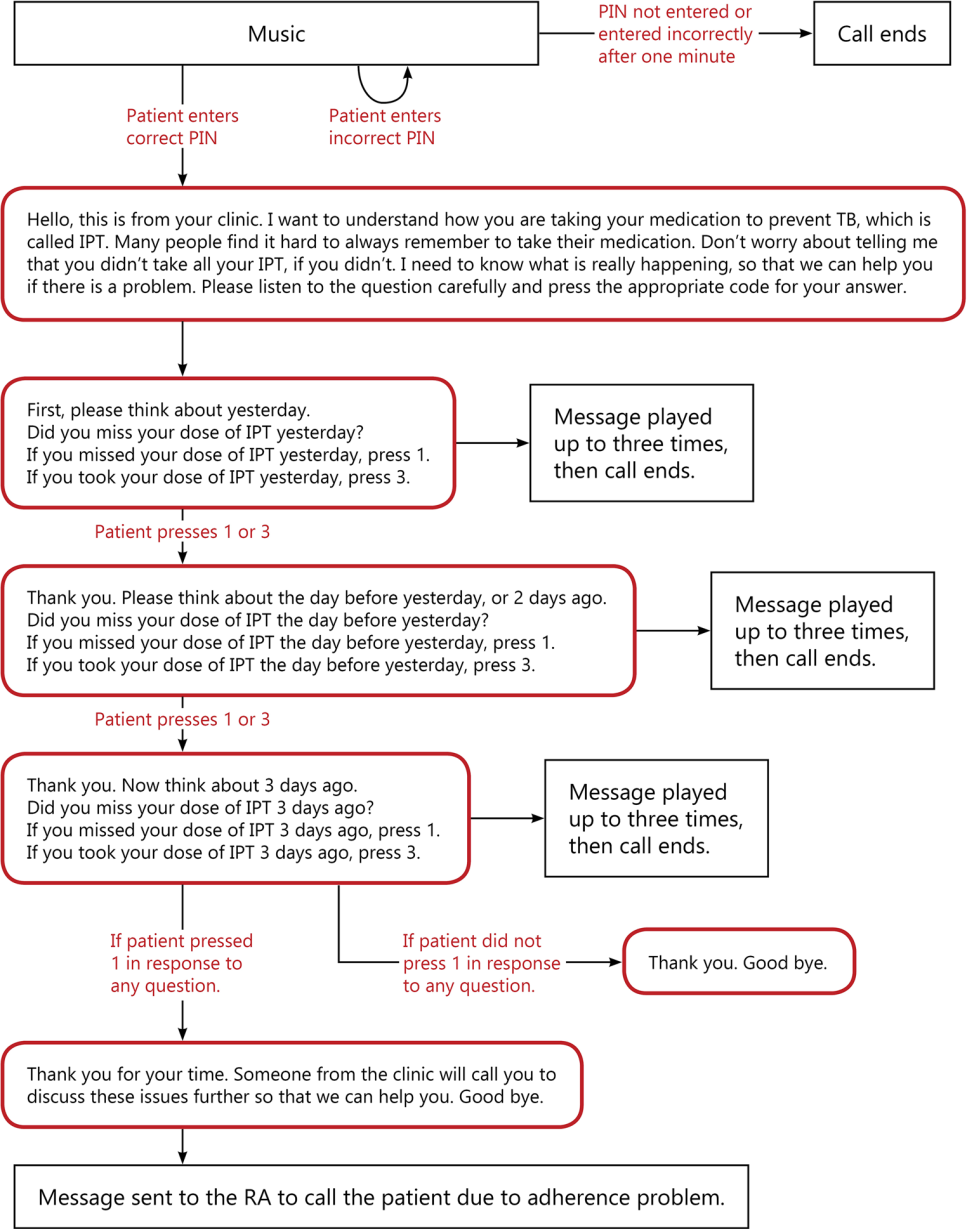
Example of IVR flow chart for medication adherence assessment call

The IVR system was designed to send four types of fully automated messages: (1) medication reminders (sent daily, and modifiable to weekly after the 1 month as per patient preferences); (2) appointment reminders (sent 1 and 2 days prior to monthly clinic visits); (3) adherence assessments (sent monthly); and (4) side effects assessments (sent monthly). To protect confidentiality, patients entered a self-selected personal identification number (PIN) in order to access and respond to any IVR message, until which time a locally popular musical melody was played. Patients were asked to key a response to all IVR messages. All IVR calls ended by thanking patients and asking if they had questions or concerns or wanted to be contacted by clinic staff. Patients who requested speaking with clinic staff were contacted by phone within 24 h. IVR calls that went unanswered were automatically re-sent 30 min later after which the call was recorded as incomplete (i.e., no answer). The IVR system generated lists of patients who did not respond to the automated messages, experienced PIN failures, reported non-adherence or side effects, or ask to be contacted, based on their keyed responses (see Figs. 1, 2 for illustrative examples of the IVR call algorithms). Patients could use the phone for personal use and to call their clinic to speak directly with clinic staff; they could also use the study SIM card and airtime with their own phone. Study staff trained patients on mobile phone use, PIN selection, and IVR call algorithms, with opportunity to practice receiving and responding to messages; patients were also counseled to take daily doses regardless of whether calls were received, in anticipation of missed calls and power or network outages. Patients chose from four available languages and selected a time to receive IVR calls, all of which were modifiable. Training was repeated a week after IPT initiation, and as needed.

### Qualitative Data Collection

Data for the qualitative evaluation were collected from May to June 2014 via interviews with 30 patient participants enrolled in the parent ENRICH Study who were enrolled at a CIP site and receiving intervention-based adherence support for IPT for a minimum of 2 weeks. We used heterogeneous sampling [21, 22] to recruit a diverse sample of patient participants, based on their gender, age, study site, and duration of exposure to IVR and IPT, to capture a broad range of issues that could influence acceptability toward IVR. The interviews were audio-recorded and privately conducted in Amharic, the most commonly spoken language, by a trained qualitative interviewer. Open-ended, exploratory questions were comprised within a semi-structured interview guide [23], and asked in casual, non-judgmental, and culturally sensitive ways to facilitate capture of participants’ perceptions and attitudes toward IVR, and perceived benefits and challenges to using IVR technology within their particular social contexts. The sequence and wording of questions changed with each interview based on participants’ individual responses to yield greater insight into their unique experiences.

### Compliance with Ethical Standards

The parent ENRICH Study and nested qualitative study received human subjects approval from the Columbia University Medical Center Institutional Review Board (Ref IRB-AAAK3163) and the National Research Ethics Review Committee in Ethiopia (Ref 3.10/780/06). All participants provided written, informed consent to participate in the parent ENRICH Study at the time of enrollment, and be interviewed monthly during IPT. As qualitative interviews were conducted with a smaller group of ENRICH participants, adjacent to a routine ENRICH Study interview, additional verbal, informed consent was sought and obtained for the qualitative interview. Verbal, informed consent was facilitated by providing participants with a detailed description of the qualitative study in print, in Amharic and read out in full by the interviewer. Participants were given adequate opportunity to ask questions about the qualitative study, and the voluntary nature of their participation was emphasized. Participants were also informed that their participation or refusal to participate in the qualitative study would have no impact on the type or quality of care they received under the ENRICH Study, and/or at their clinic. Verbal consent was recorded by the interviewer on distinct interview sheets, prior to commencement of each qualitative interview. All participants approached for the qualitative interview agreed to participate. They were each provided with a light snack and soft drink. The study adhered to COREQ guidelines [24].

### Analysis

Audio recordings from the interviews were transcribed verbatim, translated, anonymized, and thematically analyzed using a grounded theory framework [21, 25]. Transcripts were coded independently by two study investigators, who practiced critical reflexivity and crosschecked emerging codes in consultation with the interviewer to enhance inter-rater reliability and ensure that the analysis was grounded in participants’ narratives [25]. Codes were further refined and contextualized through a process of constant and discursive comparative analysis [25] to facilitate the emergence of several intersecting themes, described ahead. Data interpretations were also informed by existing understandings of patient experiences, decision-making, and m-health interventions in TB and HIV. Qualitative analysis was supplemented by the following data: participants’ socio-demographic characteristics at baseline, collected via interviewer-administered standardized questionnaires at the time of enrollment into the parent study; and their aggregate IVR usage, collected via weekly IVR logs produced by MOTECH from baseline to the point of qualitative interview.

## Findings

Interviews were completed with 16 women and 14 men (Table 1). At baseline, participants’ mean age was 33 years (range 18–58 years). The average household size was 3.2, and 50 % of participants were married or living together with their partner. Sixty-seven percent were working, 93 % reported having electricity in the household, 50 % reported a telephone in the household, and 50 % reported owning a cell phone. Seventy-three percent of participants had completed primary school. Literacy, defined as the ability to read a whole sentence, was reported by 57 % of participants. Seventeen percent of participants had not disclosed their HIV status to anyone outside of the health system.

**Table 1:** Baseline participant characteristics and summary of IVR calls information

	n = 30 N (%)
Age, y	
Mean (SD)	32.8 (9.7)
Sex	
Male	14 (46.7)
Female	16 (53.3)
ART status	
On ART	28 (93 %)
Unknown	2 (7 %)
Marital status	
Married/living together	15 (50.0)
Divorced/separated	7 (23.3)
Widowed	4 (13.3)
Never married/never lived together	4 (13.3)
Number of household members	
Mean (SD)	3.2 (2.2)
Education level	
No school	2 (6.7)
Primary school	20 (66.7)
Secondary school	6 (20.0)
Higher	2 (6.7)
Employment	
Working for cash/in-kind payment	19 (63.3)
Not working for payment	1 (3.3)
Not working at all	10 (33.3)
Electricity in household	28 (93.3)
Telephone in household	15 (50.0)
Owns a cell phone	15 (50.0)
Literacy	
Cannot read at all	7 (23.3)
Only able to read part of sentence	6 (20.0)
Able to read whole sentence	17 (56.7)
Problems learning about medical condition because of difficulty understanding written information?	
Always/often	9 (30.0)
Occasionally	1 (3.3)
Never	20 (66.7)
Disclosed HIV status to^a^	
No one	5 (16.7)
Relative	18 (60.0)
Friend	7 (23.3)
Call type	
Pill reminder	2591 (90.4)
Appointment reminder	168 (5.9)
Adherence assessment	53 (1.9)
Side effects assessment	55 (1.9)
Call outcome	
Total calls	2867
Complete	674 (23.5)
Incomplete	2193 (76.5)
Reasons for incomplete calls	
No answer	1056 (48)
Failed^b^	540 (24.6)
PIN failure	304 (13.9)
Busy	76 (3.5)
Unknown	217 (10)

^a^Multiple answers allowed

^b^Failed calls included those that were disconnected prematurely (before IVR messages were completely played) either by the participant or due to a network malfunction

Participants were proportionately distributed across the study intervention sites. At the time of interview, they had been on IPT and exposed to the IVR system for an average of 17.4 weeks (range 4–26). Of 2867 calls attempted for the 30 participants, 90 % were daily medication reminder calls, 6 % were monthly appointment reminder calls, 2 % were monthly adherence assessment calls, and 2 % were monthly side effects assessment calls. IVR calls were completely answered, with PIN entry and response to the automated message, 24 % of the time. The most common reasons that calls were designated incomplete were: no answer (48 %), premature disconnection by the network or participant (25 %), and PIN failure (14 %) (Table 1).

Four themes emerged from our data: satisfaction with automated calls, maintaining HIV confidentiality, preferences for calls versus visits, and literacy related to IVR technology.

### Satisfaction with Automated Calls

Overall, participants were enthusiastic about the IVR component of the CIP. Fifteen participants had not owned a phone, and for most of them, this was their first time interacting with mobile technology. They were grateful to have been entrusted with a device that would have otherwise remained inaccessible to them.

The various messages offered many participants a sense of “life” or “health”. Daily reminders relieved participants of the stress of having to remember to take their medication every day, and delivered a novel form of treatment support that many were unable to receive in their home or from relatives.

“What I would like and love from the phone device, it reminds me. It reminds the time when I am taking my medication and helping me not to be reluctant… I think the phone is given to me as a guarantee to my life… When I see that I am becoming well, as compared with the one I suffered, I would like to thank them… the one which is being done is secretly and nobody knows about me… When I take my pills at this time, who supported me? My families, my brothers do not support me.” 40 y.o. male

Several participants believed they would have forgotten or delayed taking their medication had it not been for the automated calls. They reported being more aware of the importance of timely treatment intake as a result of the daily reminders, and this appeared to boost their commitment to IPT. In many instances, the call itself was considered an adequate reminder even when participants did not proceed to answer the call, enter their PIN, and/or respond completely to specific messages.

“Since I can easily forget, it helps to remind me… Because of work related and as a human being, I forget. When I receive a call, I remember and take it immediately.” 27 y.o. female

“When the phone rings, I know that it is eight o’clock. Even if I forget taking my pills, while putting my phone [in my pocket] or somewhere in the house, when I listen to the ring, I will say it is eight o’clock and take my pills.” 25 y.o. female

Most participants said they had successfully integrated the IVR calls into their daily routines. They had self-selected preferred times for IVR calls (six participants opted to receive weekly, as opposed to daily medication reminders after 1 month of IPT), and many reported carrying the mobile phone on their person to avoid missing an IVR or related call from their clinic. Participants enjoyed that calls began with a musical melody that mandated PIN entry before any personal health or treatment related messages were played. Participants appreciated that PIN entry helped protect their “secrets” or the confidentiality of their illness.

“When they gave me the phone device. It has a code and nobody can pick the received call and I correctly insert the code… for example if I put my phone device and go away, anybody cannot know my secrets.” 30 y.o. male

### Maintaining HIV Confidentiality

It followed that while participants appreciated receiving a phone as part of the study intervention, it became a marker of their illness, and only a few discussed it with relatives and friends. Most participants were reticent about disclosing its purpose to others, as they were afraid it could lead to HIV disclosure.

“My friends ask me and tell them that I bought it by myself. However, I don’t tell them that I got it from here because my friends don’t know about my illness… it is a secret.” 29 y.o. male

All participants accepted the study-issued phone. However, some participants who owned a phone prior to the study reported transferring the study SIM card into their existing device to avoid arousing suspicion about a new phone.

“My relative always asks me where I got the phone and I wanted to hide from her. She is talkative. I inserted the SIM into this phone device and hide that device in my house… it is to limit the spread of rumors.” 50 y.o. female

When answering IVR calls, participants adopted diverse strategies to avoid drawing attention to themselves. They found a reason to be excused when in the company of people to whom they had not disclosed. Several participants altogether rejected IVR calls when in public.

“Every day when the call comes, I know that it is from them; even if I am with somebody, I will walk away.” 25 y.o. female

“Sometimes when somebody sat beside me, or people around me, I was scared to respond the call, and reject the call.” 23 y.o. female

At least one participant kept her phone hidden and disconnected except when she expected the IVR call. She had not disclosed her HIV status to her partner, with whom she lived, and was afraid of his reaction if he were to discover the purpose of the phone or the automated calls. Consequently, she did not respond to follow-up calls that were made outside of this specified time.

Alongside privacy concerns, participants commonly turned off their phones in order to save battery power in anticipation of power outages. Participants who said they rejected IVR calls due to a lack of privacy, or left their phone turned off or away from their person for extended periods, more often reported not receiving IVR calls consistently.

### Preferences for Calls Versus Visits

Participants had varying responses in relation to their preferences for IVR calls and in-person clinic visits. Most participants appreciated the convenience of discussing their health status over the phone, and triaging symptoms in advance of enduring the cost and time associated with an in-person visit.

“It is better to communicate via phone. Rather than coming from my house to here; transportation problem and lots of suffering while travelling.” 23 y.o. female

The ability to communicate via phone alleviated some participants’ stress of speaking directly to clinic staff or being identified by others at an HIV clinic, and having to deal with the social criticism, judgment or stigma related to such events.

“When responding via phone, nobody watches and gazes at me so the phone benefits me in this… I mean since I can get information how to use the pill while staying at home, I am not expected to come and ask the doctor how is this, what is that.” 35 y.o. male

“I can explain very well via phone device and you don’t know who I am and you only know my number. So I can talk with my problems without any fear. If it was in physical presence, it could be difficult and I am scared to talk, so I think it has this advantage.” 58 y.o. male

Participants also appreciated that the phone strengthened their capacity to communicate directly with clinic staff, of their own volition, rather than waiting to voice their concern at a scheduled visit.

“The phone is very important because it helps me to call and communicate with them when I am sick and receive calls from the clinic. Rather than totally depend on somebody, I can receive calls and I am able to call them when appropriate.” 55 y.o. female

It became clear over the course of the study that it was this capacity to speak with a health care worker, as opposed to communicate with an automated system, that drove participants’ preference towards phone-base communication.

“If I encounter side effects, for example tingling or numbness, itching, nausea I have already told her, I will rather communicate via her personal phone rather than waiting for all these numbers and respond.” 40 y.o. male

A smaller group of participants were explicit in their preference to interact with health care workers in person. They believed a clinic visit was the only way to have their problems appropriately addressed.

“It is better to go and talk to nurses than via phone. Responding via phone is good but it is better to come here and show my body. For me, it is better to speak and say I encountered this. Over the phone, I only follow the computer instruction, I cannot talk.” 38 y.o. male

Most of these participants expressed greater difficulty understanding and responding to the automated messages. They also appeared to suffer more social hardships and need a greater degree of health care worker support, compared to participants who voiced more positive experiences with the IVR system.

“There is a need of psychological morale for patients. Sometimes, as life become difficult, people lose their hope so there should be somebody to encourage them… they have to give priority to those physically weak and very sick patients.” 40 y.o. male

### Literacy Related to IVR Technology

The wide variation in participants’ literacy and understanding related to IVR technology became apparent as their responses to specific IVR messages were probed. Although they all reported being satisfied with the instruction offered on the use of the mobile phone and IVR system, very few participants demonstrated a clear understanding of the four types of IVR messages and expected responses. Participants said they failed to enter their PIN within the stated timeframe, key appropriate entries that were meant to confirm medication adherence or record experiences with side effects, and sometimes forgot their PIN. A few participants found the IVR messages to be brief, with insufficient explanation on possible responses. This was corroborated by the high proportion of IVR calls designated incomplete.

“Some of the messages might not be clear to understand… It is because when they said, ‘If you have problem press one, if not press three’, it is very short and how many people could understand this?” 58 y.o. male

“When I receive the call, I pick up the phone. I listen to, I don’t say hello but simply put the phone on my ear. After I see the classical [music] then I end the call… but I don’t know about the code.” 45 y.o. female

However, despite poor IVR literacy, participants appeared to be successfully reminded about their daily doses. “The message is the music”—that the phone rung at a consistent time and played music each day was sufficient reminder.

“I listen to the music for a couple of minutes. I hear no sound at all. It happens again and again. When the time is 8:05 or 8:06 it ends by itself… I see my watch and find out that it is the time to take my pills. In this case, I prepare myself to take my pills… The main thing is the time reminder.” 32 y.o. female

An important feature of the automated system allowed patients to request a call-back from their clinic, and have their concerns addressed over the phone in advance of scheduled visits. This emerged as the most confusing component of the IVR system, as participants who activated this feature expected to be immediately connected to a health care worker.

“When it says, ‘Press one’, when you press one, I believe, it is better to have doctors to consult… Sometimes there is a time which makes me very uncomfortable… at which time I needed a doctor and press number one but it ends the call.” 27 y.o. male

Participants’ confusion with this component of the IVR system was compounded when staff failed to follow-up call-back requests in a timely manner.

“One thing I have reservation is it says ‘If you have any questions, please press one to call to our clinic’. Then when you press one, we receive an answer, ‘You will receive a call from the clinic immediately’. However, I didn’t receive call… If I receive calls when I press one it could be very good.” 40 y.o. male

Upon experiencing difficulties with the IVR system, participants felt disinclined to have a relative or friend examine the device or listen in on calls. They did not seek clarification or retraining from clinic staff, worried that this would be an imposition on staff time. Instead, they attributed any problem with the IVR system to a network malfunction, even when it may have been due to clinic oversight or personal error.

Participants dealt with these challenges by visiting their clinic in advance of their scheduled appointment, for immediate staff attention. A few participants turned off their phones for a few days waiting for network problems to resolve. In general, though, they reported continuing to take their daily doses on time, and adhering to clinic appointments.

“I call [the nurse] and tell her that I didn’t receive calls from the clinic. She told me that it was the network. Sometimes 30 min is passed from the usual time and I said what happened to them… I do not forget, they ordered me with precautions not to discontinue the pills. Then, I by myself remember correctly besides the telephone reminder.” 30 y.o. male

## Discussion

To our knowledge, this is the first study to characterize HIV-positive patients’ acceptability toward IVR technology while receiving IPT, and to actively explore patient preferences for phone-based and in-person communication. Patients were exposed to IVR for up to 6 months, akin to patients in other studies testing the effectiveness of IVR [18, 26]. We identified several enablers to IVR use: individualized IVR call times and frequency; treatment understanding and awareness; and diminished costs and waiting times related to clinic visits. We also identified several barriers: inadequate instruction on phone and IVR use; lack of clarity on various IVR messages and prompts, including PIN entry; network malfunctions and possible power outages; lack of two-way communication between patients and providers; and delayed follow-up of automated entries on the part of health care workers. Similar problems with IVR comprehension, especially PIN entry, and a failure to implement repeated interpersonal communication have been identified as barriers to implementing phone-based interventions among TB patients in India [26] and PLHIV in Uganda [18].

**Fig. 3:**
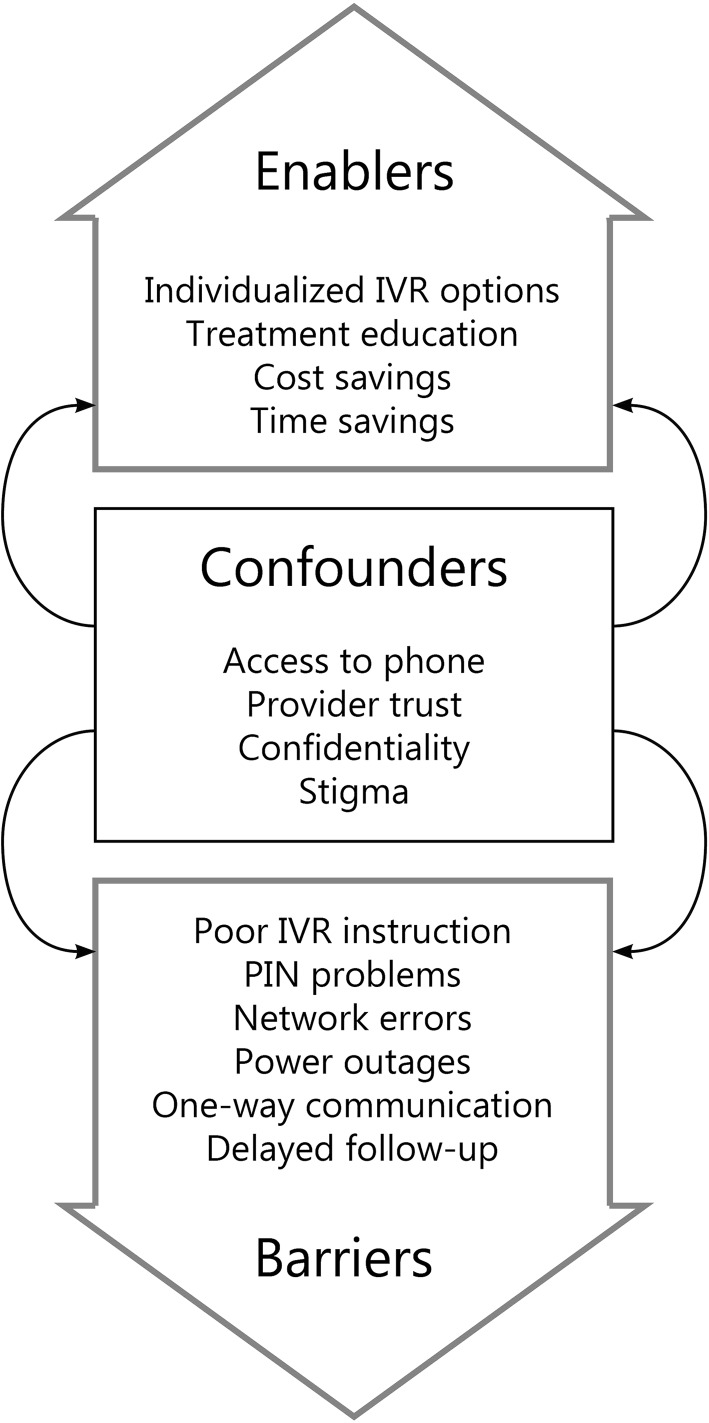
Enablers barriers, and intersecting factors influencing IVR acceptablity

We found provider trust and communication, HIV stigma and confidentiality, phone and IVR literacy, and the incentive of a new phone confounded IVR acceptability. Several participants expressed dissatisfaction with the IVR system because they were unable to directly communicate with a trusted provider. However, participants who were more familiar with mobile phones were empowered to place direct calls to providers when needed, which enhanced their acceptability toward IVR. Phone-based communication also influenced participants’ visibility within community and clinic settings in contrasting ways. For many patients, IVR calls enabled release from the stigma of being seen, labeled, and judged at an HIV clinic. On the other hand, a new device as well as IVR calls attracted unwanted attention upon patients who had not disclosed their HIV status to household members. Finally, access to a new phone—a perceived valuable commodity—enabled acceptability among patients who had not previously owned a phone. However, inexperienced phone users were also more easily frustrated by their attempts to manage automated calls. The complex ways in which IVR acceptability could be enhanced or inhibited by patients’ social and medical contexts should be considered when deploying m-health innovations among HIV-affected populations in similar high-burden settings (Fig. 3).

Among HIV-positive patients, IVR technology has primarily been used to monitor and measure changes in health behavior such as adherence to treatment [17, 18]. In our study, most IVR calls were intended to encourage rather than assess adherence to treatment. Although many of these calls were designated incomplete under the algorithm’s objective criteria, they likely served their purpose in reminding patients to take medication. Incomplete calls, however, may have failed to capture data on adherence to treatment or frequency of side effects. We thus suggest that the IVR system may be best suited as an adherence cue for treatment intake and clinic visits, and as a tool to enhance patient-provider communication, rather than to achieve more complex tasks such as monitoring the frequency and type of medication side effects. Research from Uganda has similarly found that while IVR is acceptable to patients, it is not an effective tool to assess adherence [19].

Our qualitative evaluation has some limitations. First, the purposive sample allowed us to illustrate the diversity in participant experiences with IVR. However, they may not represent the perspectives of PLHIV in settings with disparate levels of mobile phone utilization. In Ethiopia as a whole, mobile phone coverage is lower than in our sample, at 25 % [27]; acceptability may be exaggerated when patients are incentivized by access to a novel, otherwise inaccessible device. Elsewhere in Africa, mobile phone usage is relatively higher, at 70 % [27]; improved network coverage, proficiency with phone technology, and lower perceived visibility related to phone use may collectively lead to greater IVR acceptability in those settings. Second, we interviewed participants at single points in time, and at varying stages of IPT, which precluded an understanding of changes in IVR usage over time. Longitudinal analyses may allow us to gauge IVR acceptability over longer periods, and its applicability in chronic conditions. We may comprehensively analyze the IVR log data once the ENRICH Study concludes, and participants complete the full course of IPT. Third, oin an effort to mitigate bias within the broader study analyses, we did not link participants’ responses to their individual IVR logs. Disaggregated data on IVR usage and objective measures of adherence to IPT would have allowed us to validate the subjective themes presented. We hope to achieve this once the parent ENRICH Study concludes.

The study unveils important considerations for the application of mobile phone technology in high HIV-burden, resource-limited settings. First, patients receiving drug therapy for extended periods may be highly motivated to use IVR for treatment follow-up and support. The success of such technology rests in its ability to adapt to patients’ complex and dynamic social environments through protection of their privacy, most notably in the case of HIV, and flexibility in call algorithms. Second, simpler automated systems are more likely to have a sustained impact on patient behavior, given their multiple social constraints, structural deficiencies that compromise stable access to phones and cellular networks, and a general lack of familiarity with automated phone technology—though this is likely to change as we have seen in other resource-limited areas in Asia and Africa. The scalability of IVR interventions is also conceivable with rising cell phone ownership, and with time, individual cell phone provision may no longer be necessary. A majority of participants had electricity in their homes, but concerns about potential power outages were common, leading them to minimize phone use to conserve battery power. This likely reflects how patients may behave in other resource-limited settings. While ongoing costs for IVR programming may be relatively low, initial set-up costs are substantial. The findings thus urge us to consider the utility of more cost-effective tools such as missed calls (‘buzzing’ [28]) or SMS text-messaging, with emoticons in case of low literacy, given that incomplete IVR calls were considered adequate adherence cues by many participants. Third, despite the benefits of IVR-based communication and treatment monitoring, it is not surprising that patients experiencing side effects may be less satisfied with IVR and continue to endure practical inconveniences to be seen by a health care worker in person. Phone technologies in health may thus be applied to triage the timeliness and frequency of such visits, as opposed to replacing them altogether. They are also more likely to be acceptable to patients who are in stable health, familiar with mobile phone technology, and more comfortable or open about their disease status.

## Conclusion

This qualitative evaluation highlights important enablers and barriers to IVR implementation from the perspective of HIV-positive patients in Ethiopia. The complexity of these determinants offers a gateway for future examination of the suitability and feasibility of m-health innovations in resource-limited settings. The findings were used to enhance intervention delivery at the study sites. Staff were trained to administer more interactive and user-friendly IVR refresher training sessions for patients placed on IPT, track and respond to patients’ requests for call-backs in a timely manner, show greater empathy and sensitivity toward patients who encountered difficulties with responding to automated messages, and counsel patients on innovative and tailored strategies to manage HIV disclosure and confidentiality within the contexts of their social realities. It is critical that we adopt such integrated approaches to knowledge production in the field of implementation science, and translate the lessons learned to guide delivery of health care services.
